# Investigating the Association Between Polygenic Risk Scores for Alzheimer’s Disease With Cognitive Performance and Intrinsic Functional Connectivity in Healthy Adults

**DOI:** 10.3389/fnagi.2022.837284

**Published:** 2022-05-11

**Authors:** Aliaa Ibnidris, Fabian Fußer, Thorsten M. Kranz, David Prvulovic, Andreas Reif, Johannes Pantel, Emiliano Albanese, Tarik Karakaya, Silke Matura

**Affiliations:** ^1^Faculty of Biomedical Sciences, Università della Svizzera Italiana, Lugano, Switzerland; ^2^Department of Psychiatry and Mental Health, University of Cape Town, Cape Town, South Africa; ^3^Department of Gerontopsychiatry, Psychosomatic Medicine, and Psychotherapy, Pfalzklinikum, Klingenmünster, Germany; ^4^Department of Psychiatry, Psychosomatic Medicine and Psychotherapy, University Hospital, Goethe University Frankfurt, Frankfurt, Germany; ^5^Institute of General Practice, Goethe University Frankfurt, Frankfurt, Germany

**Keywords:** Alzheimer’s disease (AD), genetic disposition, polygenic risk score (PRS), cognition, intrinsic functional connectivity, resting-state fMRI

## Abstract

**Background:**

Alzheimer’s disease (AD) pathology is present many years before the onset of clinical symptoms. AD dementia cannot be treated. Timely and early detection of people at risk of developing AD is key for primary and secondary prevention. Moreover, understanding the underlying pathology that is present in the earliest stages of AD, and the genetic predisposition to that might contribute to the development of targeted disease-modifying treatments.

**Objectives:**

In this study, we aimed to explore whether genetic disposition to AD in asymptomatic individuals is associated with altered intrinsic functional connectivity as well as cognitive performance on neuropsychological tests.

**Methods:**

We examined 136 cognitively healthy adults (old group: mean age = 69.32, SD = 4.23; young group: mean age = 31.34, SD = 13.12). All participants had undergone resting-state functional magnetic resonance imagining (fMRI), DNA genotyping to ascertain polygenic risk scores (PRS), and neuropsychological testing for global cognition, working memory, verbal fluency, and executive functions.

**Results:**

Two-step hierarchical regression analysis revealed that higher PRS was significantly associated with lower scores in working memory tasks [Letter Number Span: Δ*R*^2^ = 0.077 (*p* < 0.05); Spatial Span: Δ*R*^2^ = 0.072 (*p* < 0.05)] in older adults (>60 years). PRS did not show significant modulations of the intrinsic functional connectivity of the posterior cingulate cortex (PCC) with other regions of interest in the brain that are affected in AD.

**Conclusion:**

Allele polymorphisms may modify the effect of other AD risk factors. This potential modulation warrants further investigations, particularly in cognitively healthy adults.

## Background

Alzheimer’s disease (AD) is a neurodegenerative disorder that is characterized by a progressive decline in cognitive function (McKhann et al., 2011). The neuropathological hallmark of AD in the brain is the presence of extracellular Aβ amyloid plaques and intracellular neurofibrillary tangles (NFT; Braak and Braak, 1991). Previous evidence shows that specific biomarker abnormalities consistent with these neuropathological changes are detectable years before the commencement of clinical symptoms (Jack et al., 2010; Sperling et al., 2011). The United States National Institute on Aging – Alzheimer’s Association (NIA-AA) proposed a biological definition of AD, allowing for the diagnosis of AD in the presence of β-amyloid and neurofibrillary tau in cognitively healthy elders (Jack et al., 2010, 2018; Albert et al., 2011; McKhann et al., 2011; Sperling et al., 2011). Because irreversible cognitive dysfunction is caused by neuronal cell death, network dysfunction and eventual neurodegeneration, it is critical to identify people at risk before this occurs. In order to design disease-modifying drugs for AD, it is widely accepted that treatments should be administered as early as possible before clinical symptoms have appeared, and ideally, earlier than the start of neuronal damage (Jessen et al., 2014, 2018). The NIA-AA criteria are not meant for clinical uses, but only for research purposes and in clinical research settings.

Resting state functional magnetic resonance imaging (rs-fMRI) may be a viable biomarker to detect altered intrinsic functional connectivity in people at risk of AD (Sorg et al., 2007). Rs-fMRI shows the intrinsic functional connectivity between brain regions at rest when no task is being performed. Previous evidence suggests that AD may be conceived as a disconnection syndrome, both structural and functional (Delbeuck et al., 2003; Sorg et al., 2007). The Default Mode Network (DMN), a set of brain regions that shows functional activity during rest, is one of the most widely studied functional networks in AD (Raichle et al., 2001; Krajcovicova et al., 2014). The Posterior Cingulate Cortex (PCC) is the posterior anatomical/computational hub in the DMN and brain in general (Hagmann et al., 2008; Greicius et al., 2009). It is suggested that the neuropathological changes and the resulting structural lesions in the brain may be associated with alteration in intrinsic brain activity in AD in the DMN (Buckner and Vincent, 2007). Previous studies show that the PCC is especially susceptible to the deposition of amyloid plaques in AD (Sperling et al., 2009; Mormino et al., 2011). Evidence from fluorodeoxyglucose positron emission tomography (FDG-PET) studies demonstrate diminished resting state glucose metabolism in the PCC of patients with early AD or MCI as well as in cognitively healthy older adults at risk of AD (Ishii et al., 2003; Buckner et al., 2005). This might reflect a possible hypometabolism or synaptic dysfunction in this region (Fessel, 2021). Previous studies investigating the functional connectivity using functional MRI observed decreased connectivity between the PCC and the hippocampus, both in MCI and AD (Greicius et al., 2004; Krajcovicova et al., 2014). On the other hand, increased connectivity is observed in the anterior DMN and in the hippocampal-medial prefrontal and the frontoparietal connectivity in similar groups (Zhang et al., 2009; Zarei et al., 2013). However, alterations in the DMN alone could only differentiate between healthy controls and people with AD but not between the different prodromal stages of AD where cognitive performance may be still preserved (Teipel et al., 2018). Because of its important role in the DMN and its vulnerability toward AD pathology, the PCC is an ideal candidate region for investigating associations between intrinsic functional connectivity and its relation to other biomarkers and risk factors of AD.

Besides alterations in intrinsic functional connectivity, there are several candidate genes that constitute another frontier for early detection of people at risk of AD. The Apolipoprotein E gene on chromosome 19 is the most commonly associated genetic risk factor for late-onset AD (LOAD), and its ε4 allele is most commonly associated with LOAD (Belloy et al., 2019; Chaudhury et al., 2019). Previous research suggests that people with MCI due to AD who were carriers of ApoE ε4 allele indeed showed altered functional connectivity as well as lower cognitive performance compared to healthy controls (Wang et al., 2015; Harrison et al., 2016). In an earlier study that investigated the relationship between resting state connectivity and genetic risk, carriers of the ApoE ε4 allele were found to have a higher activation across several cortical regions (Bookheimer et al., 2000). However, the study only looked at carriers of AD-related, ApoE variants, and investigated connectivity alterations on task-based fMRI where participants were asked to perform a memory-activation task that is sensitive to the identification of neuropathological changes in the medical temporal lobe (MTL) structures (Bookheimer et al., 2000). Nonetheless, despite its strong genetic association with AD, clinical trials for disease-modifying treatments targeting the amyloid cascade pathway and focusing on carriers of ApoE ε4 did not yield successful results yet (Yiannopoulou et al., 2019; Serrano-Pozo et al., 2021). Moreover, the relationship between specific alterations of functional connectivity on rs-fMRI should not be attributed to a single gene and further investigation of the impact of other genetic variants should be considered (Harrison and Bookheimer, 2016).

This has directed the focus on investigating other pathways and other possible genetic variants associated with AD. Genome Wide Association Studies (GWAS) have identified several single nucleotide polymorphisms (SNPs) that are associated with an increased risk of developing AD in late life (Baker and Escott-Price, 2020). These include CLU, PICALM, and CR1 as well as BIN1, ABCA7, and EPHA1 (Harrison and Bookheimer, 2016). Homozygous carriers of CLU, for example, show a stronger magnitude of intrinsic functional connectivity compared to non-carriers (Zhang et al., 2015). A suggested approach would be to investigate the effect of these SNPs, combined in a polygenic risk score (PRS), as a biomarker to reliably detect an elevated risk of developing AD already in its earliest stages. A PRS is a method to predict the genetic susceptibility of an individual to a specific disease by its summarized genetic risk for the disease based on previous evidence, and that can be used for clinical prediction rules in conjunction with the clinical history and physical examination. However, consistent evidence on the effect of other genetic variants, calculated in a PRS, on intrinsic functional connectivity in cognitively healthy older adults at risk of AD is still lacking. Therefore, the association between alterations in functional connectivity and a PRS that combines the summed and weighted risk of several genetic variants in one metric would increase the prediction power for people at higher risk of developing AD dementia.

The aim of this study was to explore whether a higher PRS in cognitively healthy adults is associated with modulation of intrinsic functional connectivity between the PCC and other regions of interest (ROIs) in the brain. We examined this association using PRS and rs-fMRI data in a cohort of healthy adults. We also aimed to examine the association of PRS with cognitive performance in validated neuropsychological tests. We hypothesized that individuals with a higher PRS show altered intrinsic functional connectivity between the PCC and other brain regions that are implicated in AD (i.e., medio-temporal lobe, MTL) as well as lower cognitive performance.

## Materials and Methods

### Participants

We examined a subset of 136 cognitively healthy participants that met the inclusion criteria for the purpose of this analysis (having rs-FMRI data, PRS, and cognitive scores available). The sample was divided into two groups with the young group including participants aged 60 years and younger (*n* = 80). 55% of the young group were females (*n* = 44). The mean age of the young group was 31.34 (SD = 13.12). The old group included those who were above 60 years of age (*n* = 56). The mean age for the old group was 69.32 (SD = 4.23). 60.71% of the old group were females (*n* = 34). Participants were drawn from a cohort of participants from the project B4 of the Neuronal Coordination – Research Focus Frankfurt (NeFF) titled “Funktionelle und strukturelle neuronale Diskonnektion als Grundlage früher episodischer Gedächtnisstörungen der Alzheimer-Krankheit” (“Functional and structural neuronal disconnection as a basis/prerequisite for early neuronal memory dysfunction in Alzheimer’s Disease”) (Matura et al., 2014a,b, 2016, 2020, 2021). The project was performed at the Laboratory for Neuroimaging of the Department of Psychiatry, Psychosomatic Medicine and Psychotherapy at the Goethe University Frankfurt, Frankfurt am Main, Germany. The methodology of this project has been extensively described in previous publications (Matura et al., 2014a,b, 2016, 2020, 2021). None of the participants had a history of neurological or psychiatric disorders. Eligible participants were selected based on the presence of a PRS and fMRI data. Sociodemographic variables of the included participants were ApoE status, age, gender, education, family history of Alzheimer’s disease dementia, handedness, weight, height, Body Mass Index (BMI), and smoking status. For the purpose of the current study, we only analyzed the association of PRS, in younger and older participants, with cognitive performance and intrinsic functional connectivity with the PCC as a seed region. The Ethics Committee of the Medical Faculty of the Goethe University Frankfurt approved the study, and all subjects signed a written informed consent. The study was undertaken in accordance with the Code of Ethics of the World Medical Association (Declaration of Helsinki) (Rickham, 1964).

### Neuropsychological Measures

We used the Mini-Mental State Examination (MMSE) to assess general cognition (Folstein et al., 1975), and the German version of the California Verbal Learning Test (CVLT) for verbal learning and short-term memory (Delis et al., 1987; Niemann et al., 2008). Furthermore, measures of working memory and attention were obtained using the Trail Making Test – Part A (Spreen and Strauss, 1998), Letter Number Span (Gold, 1997), and the spatial Span test of the Wechsler Memory Scale (Wechsler, 1997). Verbal fluency was tested using two subsets of the CERAD-NP (Consortium to Establish a Registry for Alzheimer’s Disease) – the semantic fluency and phonemic fluency tests (Morris et al., 1988). Finally, we used the Memory Complaint Questionnaire (MAC-Q) to assess subjective memory decline, (Crook et al., 1992).

### Magnetic Resonance Imagining Hardware and Procedure

All details about the study design and methods have been previously reported (Matura et al., 2014a,b, 2016, 2020, 2021). All MR images were acquired using a Trio 3-T scanner (Siemens, Erlangen, Germany) with a standard head coil for radiofrequency transmission and signal reception. Participants were outfitted with protective earplugs to reduce scanner noise. For T1-weighted structural brain imaging, an optimized 3D modified driven equilibrium Fourier transform sequence (Deichmann et al., 2004) with the following parameters was conducted: acquisition matrix = 256 × 256, repetition time (TR) = 7.92 ms, echo time (TE) = 2.48 ms, field of view (FOV) = 256 mm, 176 slices and 1.0-mm slice thickness. Functional resting state images were acquired using a blood oxygen level-dependent-sensitive echo-planar imaging sequence comprising the following parameters: 300 volumes, voxel size: 3 mm × 3 mm × 3 mm, TR = 2000 ms, TE = 30 ms, 30 slices, slice thickness = 3 mm, distance factor = 20%, flip angle = 90°, and FOV = 192 mm. Resting state measurements were part of a larger fMRI study on episodic memory. For the resting state measurements, all participants were instructed to keep their eyes open, to lie still, not to engage in any speech, to think of nothing special and to look at a white fixation cross-presented in the center of the visual field during the whole scan procedure.

### Resting-State Functional Magnetic Resonance Imagining Data Analysis

To analyze the resting state functional data, we used the Connectivity (CONN) Toolbox (CONN functional connectivity toolbox, 2012). CONN is an open-source Matlab/SPM-based cross-platform software for the computation, display, and analysis of functional connectivity Magnetic Resonance Imaging (fcMRI). CONN is used to analyze resting state data (rs-fMRI) as well as task-related designs. We first imported the raw/partially processed Digital Imaging and Communications in Medicine (DICOM) functional and anatomical files into the CONN graphical user interface (GUI). We then ran the default pre-processing pipeline (direct normalization to MNI-space) in CONN’s GUI. The pipeline performs the following steps:

– Functional realignment using SPM12 realign and unwrap procedure (Andersson et al., 2001).
– Correction of temporal misalignment of slices of functional data using SPM12 slice-timing correction (STC) procedure (Henson et al., 1999).
– Outlier identification from the observed global BOLD signal and the amount of subject-motion in the scanner.
– Direct segmentation and normalization into standard MNI space and segmented into gray matter, white matter, and CSF tissue classes using SPM12 unified segmentation and normalization procedure (Ashburner and Friston, 2005).
– Functional smoothing using spatial convolution with a Gaussian kernel of 8 mm full width half maximum (FWHM).

After pre-processing was completed, we ran the default denoising pipeline in CONN. The pipeline performs two general steps: linear regression of potential confounding effects in the BOLD signal, and temporal band-pass filtering. Once completed, we evaluated the effect of denoising by assessing the CONN Quality Control Plots. These plots provide a visualization of the distribution of functional connectivity values (FC) between randomly selected pairs of points within the brain before and after denoising. After denoising, FC distributions showed approximately centered distributions, with small but noticeable larger tails in the positive side, and considerably reduced inter-session and inter-subject variability.

### Selection of Seed Region

To analyze DMN connectivity, we used a seed region-based approach. Because we were specifically interested in DMN resting state activity and whether any alteration is associated with the PRS we investigated the intrinsic functional connectivity of a region anatomically co-localized with the major posterior hub of the DMN, the posterior cingulate cortex (PCC). We also explored the connectivity of the PCC as the region-of-Interest (ROI) with other ROIs in the brain that are commonly affected by AD pathology such as the bilateral hippocampus, bilateral anterior and posterior parahippocampus, and bilateral amygdala.

### DNA Extraction, Genome-Wide Genotyping and Polygenic Risk Scores Calculation

DNA was extracted from whole-blood samples. The DNA extraction and genotyping process were conducted at bio.logis laboratories (Frankfurt am Main, Germany). DNA was genotyped on the Infinium Global Screen Array (GSA) with multi-disease drop in (MD) covering in total ca. 700K SNPs per person at Broad Institute, Cambridge, MA, United States. For PRS calculation, 177 persons were originally considered, after quality control measures using PLINK v1.9 (Chang et al., 2015) regarding relatedness and missingness per individual (<0.1), 142 participants remained for further analysis and 136 met the inclusion criteria mentioned above and were included in this study. Regarding SNP quality, SNPs were filtered excluding minor allele frequencies (MAF < 0.01) and genotyping missing rate per marker (<0.05), deviations from Hardy Weinberg Equilibrium (1 × e^–6^) and deviations from heterozygosity. A principal component and ancestry analysis revealed that the sample is of predominantly CEU ancestry.

Polygenic risk scores were calculated using the PRSice software version 2.3.1.e with default options (clump-kb 250, clump-p 1.0, clump r^2^ 0.1 stat BETA) (Choi and O’Reilly, 2019). The summary statistics stage 1 from the International Genomics of Alzheimer’s Project (IGAP) were used (Kunkle et al., 2019) and subjected to INFO score filtering (INFO > 0.8). Neither the present study sample nor the IGAP sample show any overlap. The ApoE region (Chr19:45,116,911–46,318,605) was excluded from the analysis due to its reported large effect size. PRS values with p-threshold (pT) of 0.1 were used for further statistical analysis since this pT explains the highest variance and provides the best prediction accuracy according to a study conducted by Leonenko et al. (2021). We performed the analysis using PRS that excluded ApoE region as the main explanatory variable. To enhance readability, we refer to this variable as PRS in this manuscript.

### Statistical Analysis

All statistical analyses for the association between PRS and neuropsychological test scores were conducted using RStudio (RStudio Team, 2020). Cognitive scores and age followed a bimodal distribution. Therefore, after conducting the analysis of cognitive performance in the whole sample, we repeated the analysis for young and old groups separately. To investigate the relationship between PRS and cognitive performance, two step hierarchical regression analyses were performed with different cognitive test scores as dependent variables in the whole sample and for the old and young age group separately. ApoE status, age, gender, and education were entered at stage one of the regression to control for these influencing variables. The PRS was entered at stage two to determine the true correlation between PRS and cognitive performance controlling for the effect of potential influencing factors. For linear regression analysis, a positive beta value reflected a positive relationship while a negative beta value reflected an inverse relationship between the variables of interest.

To examine the association of PRS and connectivity values of the included participants, we performed an analysis of the whole group using multiple linear regression in the CONN Toolbox and controlled for ApoE status, age, gender and education. Functional connectivity values at rest between the PCC as the seed region and other ROIs were encoded as the dependant variable. To verify the results, the same analysis was conducted in R studio, using the beta values of connectivity between the PCC and the ROIs as the dependant variables, PRS as the explanatory variable, and controlled for ApoE status, age, gender and education.

## Results

### Participants’ Demographic Characteristics

Table 1 describes the demographic characteristics and neuropsychological tests scores of the included participants. The final sample size was 136 participants. 57% of all participants were female (*n* = 78). The mean age of the young group was 31.34 years (SD = 13.12) while for the old group the mean age was 69.32 years (SD = 4.23).

**TABLE 1:** Demographic characteristics and neuropsychological tests scores of the study sample.

Variable	Young group (*n* = 80)	Old group (*n* = 56)
Age	31.34 (13.12)	69.32 (4.23)
Gender (% female)	44 (55%)	34 (60.71%)
Years of education	16.71 (3.16)	15.33 (3.14)
MMSE	29.33 (1.54)	29.05 (1.10)
CVLT immediate recall trial 1 list A	65.62 (7.21)	55.72 (10.24)
CVLT immediate recall trial 1 list B	8.19 (2.17)	5.66 (2.04)
CVLT total immediate recall list A	9.46 (2.39)	7.21 (2.23)
CVLT short delayed free recall	14.06 (2.14)	11.30 (3.10)
CVLT short delayed cued recall	14.35 (1.91)	12.42 (2.48)
CVLT long delayed free recall	14.46 (2.01)	12.19 (3.25)
CVLT long delayed cued recall	14.65 (1.73)	12.47 (2.84)
CVLT Intrusion	1.79 (2.41)	3.29 (4.52)
CVLT recognition discriminability	15.69 (0.65)	14.96 (1.39)
TMT – A	23.86 (7.76)	39.21 (10.02)
CERAD semantic fluency (Animals)	26.53 (3.48)	22.52 (4.97)
CERAD phonemic fluency (s-words)	15.60 (3.64)	15.50 (4.62)
Letter number span	17.41 (2.77)	14.91 (2.71)
Spatial span	18.54 (2.79)	15.48 (2.79)
MAC-Q	26.07 (2.97)	26.47 (4.19)

*Values are presented by mean of raw values ± standard deviation (SD) unless stated otherwise. MMSE, Mini-Mental State Examination; CVLT, California Verbal learning Test; TMT_A, Trail Making Test – Part A; CERAD, Consortium to Establish a Registry for Alzheimer’s Disease; MAC-Q, Assessment of Memory Complaint Questionnaire.*

### Relationship Between Polygenic Risk Scores and Intrinsic Connectivity

After controlling for ApoE status, age, gender and education, seed-based correlation and ROI-to-ROI analysis of a pooled group of both young and old adults (*n* = 136) revealed no significant association between the PRS and intrinsic functional connectivity of the PCC with other ROIs (q-FDR > 0.05). More specifically, we looked at the connectivity of the PCC with largely implicated regions in AD pathology that are critical for episodic and spatial memory [i.e., the medial temporal lobe (MTL)] and whose functional connectivity is also altered in early AD (Cutsuridis and Yoshida, 2017; Berron et al., 2020). Our results did not show a significant association between the connectivity of the PCC and bilateral hippocampus in participants with PRS (right: beta = 53.80, q-FDR = 0.930829; left: beta = −54.82, q-FDR = 0.927068). Moreover, there was no significant association of the connectivity between both the right posterior parahippocampus (beta = −109.68, q-FDR = 0.920784) and the left posterior parahippocampus (beta = 79.07, q-FDR = 0.920784) and the PCC with individual PRS. We furthermore explored alterations of connectivity of the PCC with the amygdala and its association with PRS and could neither find significant results for the right amygdala (beta = 36.25, q-FDR = 0.985057), nor for the left amygdala (beta = 65.50, q-FDR = 0.985057).

To verify the results, we extracted the beta connectivity values of the PCC and the above-mentioned regions and examined their association with the respective PRS of each individual in RStudio using multiple linear regression. This additional analysis did not yield any significant association between the PRS and PCC connectivity with other ROIs, neither in the young nor in the old group (*p* > 0.05).

### Relationship Between Polygenic Risk Scores and Cognitive Performance of Young and Old Groups

**FIGURE 1:**
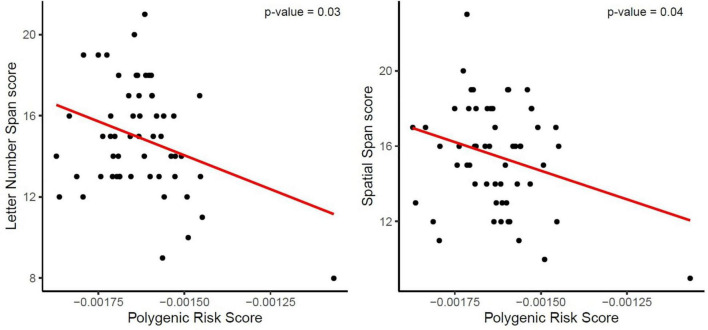
Association between the Polygenic Risk Score (PRS) for AD and performance in working memory tests (Letter Number Span and Spatial Span). The red line demonstrates that a higher genetic risk for AD, reflected in a higher PRS, is associated with decreased working memory capacity, reflected in lower scores, in both tests.

The two-step hierarchical regression revealed PRS to be a significant predictor for cognitive performance in Letter Number Span and Spatial Span in elderly individuals (>60 years). The regression model with Letter Number Span as dependent variable revealed that at stage one, gender, age, education, and ApoE status did not contribute significantly to the regression model [*F*(4,50) = 2.06, *p* = 0.10] and accounted for 14.2% of the variation in Letter Number Span scores. Introducing the PRS ratio explained an additional 7.7% of variation in Letter Number Span Scores and this change in R^2^ was significant [*F*(5,49) = 2.74, *p* = 0.029]. Together the five independent variables accounted for 21.9% of the variance in Letter Number Span scores (see Table 2). The regression model with Spatial Span as dependent variable revealed that at stage one, gender, age, education, and ApoE status did not contribute significantly to the regression model [*F*(4,50) = 2.42, *p* = 0.06] and accounted for 16.2% of the variation in Spatial Span scores. Introducing the PRS explained an additional 7.2% of variation in Spatial Span Scores and this change in R^2^ was significant [*F*(5,49) = 3.00, *p* = 0.019]. Together the five independent variables accounted for 23.4% of the variance in Spatial Span scores (see Table 3). The association between PRS and performance in working memory tests (Letter Number Span and Spatial Span) in the old group is demonstrated in Figure 1. Statistical analyses with the whole group did not reveal any significant associations between PRS and cognitive performance. Furthermore, there were no significant associations between PRS and cognitive performance in the group of young individuals.

**TABLE 2:** Summary of hierarchical regression analysis for variables predicting Letter Number Span performance.

Variable	*B*	SE *B*	β	*T*	*p*
Step 1					
ApoE	0.12	0.28	0.057	0.43	0.67
Age	−0.13	0.08	−0.20	−1.52	0.14
Gender	0.82	0.76	0.15	1.08	0.28
Education	0.19	0.12	0.22	1.60	0.11
Step 2					
ApoE	0.15	0.27	0.07	0.55	0.58
Age	−0.11	0.08	−0.17	−1.33	0.19
Gender	0.96	0.73	0.17	1.30	0.20
Education	0.16	0.12	0.18	1.38	0.17
PRS	−6073.0	2763.1	−0.28	−2.19	0.03

*R^2^ = 0.142 for Step 1, ΔR^2^ = 0.077 (p < 0.05).*

**TABLE 3:** Summary of hierarchical regression analysis for variables predicting Spatial Span performance.

Variable	*B*	SE *B*	β	*T*	*p*
Step 1					
ApoE	0.56	0.28	0.057	0.19	0.84
Age	−0.14	0.09	−0.20	−1.64	0.11
Gender	1.47	0.76	0.15	1.90	0.06
Education	0.18	0.12	0.22	0.96	0.34
Step 2					
ApoE	0.09	0.28	0.04	0.31	0.76
Age	−0.12	0.08	−0.18	−1.45	0.15
Gender	1.61	0.75	0.28	2.14	0.04
Education	0.08	0.12	0.09	0.72	0.47
PRS	−6093.3	2815.3	−0.27	−2.15	0.04

*R^2^ = 0.162 for Step 1, ΔR^2^ = 0.072 (p < 0.05).*

## Discussion

In a cohort of cognitively healthy adults, we conducted an exploratory analysis to investigate the association between the genetic risk for AD reflected in a PRS and intrinsic functional connectivity. Our findings demonstrate that PRS did not have a significant predictive effect on the intrinsic functional connectivity of the PCC with other regions in the brain that are implicated in AD. In addition, we investigated the effect of PRS on cognitive performance. Our results show that a higher PRS seems to have a significant association with working memory performance in participants older than 60 years. In this group, a higher PRS was significantly associated with worse performance in tests of working memory capacity. In the next paragraphs, we aim to contextualize our findings, and to interpret their implication in practical terms as well as account for the limitations of the present study.

Previous evidence suggests that the DMN and the PCC connectivity are greatly disrupted in AD by amyloid deposition in DMN regions (Greicius et al., 2004; Sperling et al., 2009; Beason-Held, 2011; Binnewijzend et al., 2012; Damoiseaux et al., 2012; Palmqvist et al., 2017). Altered functional connectivity of the DMN has also been observed in cognitively healthy ApoE ε4 carriers as well as in patients with MCI or AD (Lambert et al., 2013). Nonetheless, clinical trials targeting the amyloid cascade hypothesis have shown inconsistent results so far (Yiannopoulou et al., 2019; Walsh et al., 2021). Anti-amyloid monoclonal antibody treatments such as the recently approved Aducanumab showed reduction in amyloid load in people with MCI or early AD (Sevigny et al., 2016; Office of the Commissioner, 2021; Walsh et al., 2021). However, the clinical benefits (i.e., reversal or slowing of cognitive decline) of this drug are yet to be confirmed in further post-approval clinical trials (Office of the Commissioner, 2021). The previous inconsistent results of clinical trials have directed research into looking for other potential genetic risk factors that are linked to AD. We first investigated whether an elevated genetic risk for AD in healthy persons, reflected in a polygenic risk score that excludes the ApoE region, may have similar alterations in intrinsic functional connectivity to that observed in patients with MCI and AD. This was done by calculating a PRS based on Single Nucleotide Polymorphisms (SNPs) that are significantly associated with AD. Results from our sample could not demonstrate a significant association between individual PRS and intrinsic functional connectivity of the PCC and other regions in the brain, namely bilateral hippocampus, bilateral anterior and posterior parahippocampus, and bilateral amygdala.

Secondly, we looked at the effect of PRS on cognitive performance. We found that a higher PRS was negatively associated with performance in tests of working memory. However, this was true only in participants who were older than 60 years. Working memory is mostly described as a system that maintains and stores information in the short term. Contents within working memory are actively held and manipulated over a limited period of time (Baddeley, 2000). Information is stored between 15 and 30 s in working memory, although this time can be substantially prolonged when the information is constantly rehearsed (Baddeley, 2000). Following Baddeley’s (2000) model, working memory can be subdivided by two main components: the phonological loop and the visuo-spatial sketchpad. The phonological loop stores phonological information and prevents its decay by continuously articulating its contents. The visuo-spatial sketchpad handles visual and spatial information. Working memory facilitates planning, comprehension, reasoning, and problem-solving (Baddeley, 2005). Previous studies showed that working memory is sensitive to AD pathology (Saunders and Summers, 2011) and that tests of working memory such as Letter Number Span can reliably differentiate healthy controls from patients with MCI or AD (Kessels et al., 2011). Some findings suggest a possible effect of age on working memory capacity with significant decline starting at the age of 60 years (Dobbs and Rule, 1989). A higher genetic risk for AD might amplify the age-related decline. Overall, there is increasing evidence that a higher genetic risk of AD reflected in a higher PRS is associated with a faster decline in memory (Ge et al., 2018; Gustavson et al., 2022). Ge et al. (2018) investigated the influence of a genetic risk for AD on Amyloid β (Aβ) accumulation in the brain, cognitive performance, and hippocampal volume over the course of 2 years. The study found that higher polygenic risk was strongly associated with higher rates of decline in memory and executive function. This was shown primarily in individuals with a cerebral accumulation of Aβ. Our finding of a negative association of PRS and memory performance in elderly participants, but not in younger individuals, could be due to the fact that there was some degree of Aβ accumulation in the brains of the elderly individuals that was not present in the younger group. Cerebral Aβ accumulation associated with Alzheimer’s Disease usually starts later in life, around the age of 60–70 years (Bilgel et al., 2016). Since we only found an association between PRS and working memory performance in the older group (<60 years), a possible explanation could be the prevalence of cerebral Aβ in this group, which may be contributing to the association between working memory performance and PRS (Dobbs and Rule, 1989). However, since we do not have information on cerebral Aβ accumulation for the participants of our study, this hypothesis remains speculative.

We conducted our analysis with a PRS that excluded the ApoE region. Previous evidence suggests that PRS excluding ApoE region is able to differentiate between cognitively healthy adults and those with amnestic MCI (who had higher PRS) (Logue et al., 2019). Findings regarding the association of PRS (including ApoE) with cognition have been inconsistent with some studies showing a lack of significant association with overall cognitive performance (Gui et al., 2014; Bressler et al., 2017), while others found PRS to be associated with a decline in working memory in cognitively healthy adults (Andrews et al., 2016) and in global cognition (Mormino et al., 2016). Our findings suggest that a higher genetic risk of AD beyond ApoE status may drive similar mechanisms that lead to cognitive impairment and deficits in working memory (Becker, 1988; Kessels et al., 2011; Saunders and Summers, 2011).

A major limitation of this present study is the small sample size, specifically after stratification of the sample by age. Since both age and cognitive scores followed a bimodal distribution, we decided to conduct the analysis of cognitive performance in two different age groups. We consider our study to be an exploratory study to examine the association of genetic risk with intrinsic connectivity and cognitive performance. Since genetic variables usually have a small effect size, the small sample size could have resulted in negative findings due to a lack of statistical power. With a larger sample size and thus more statistical power we might have been able to also detect an effect of genetic risk for AD on intrinsic functional connectivity. Studies with small sample sizes have the advantage of allowing to investigate such association while using feasible resources (e.g., less of the participants’ time and lower financial costs). Nonetheless, larger studies are required to further examine and confirm results generated from studies with smaller sample size.

Another limitation is that we only investigate the association of the PRS and intrinsic functional connectivity in cognitively healthy adults. We did not compare this cohort of participants to a group of patients with MCI or AD. Therefore, we could not compare the results and identify potential similarities or differences between the two groups. However, our results still provide a considerable insight into the potential role of genetic variants of AD beyond ApoE ε4 on the modulation of cognitive functions. As this was conducted as an exploratory analysis, further investigation, and comparison between cognitively healthy and people with MCI/AD is imperative to compliment the presented findings. Another potential limitation is the unavailability of longitudinal data of the analyzed sub-sample of this cohort. We analyzed the functional connectivity data that was taken at a cross-sectional point in time. Insightful information could be gathered if we follow up this sample and investigate whether there is further significant modulation in the intrinsic functional connectivity or cognitive decline in relation to their PRS. Secondly, carriers of genetic variants associated with AD may not necessarily express the phenotype (i.e., typical clinical symptoms of AD) in their lifetime. Therefore, it is important to track those who have shown a worse performance in working memory tasks and examine whether they started to express clinical symptoms, show alteration in intrinsic functional connectivity, and develop further cognitive decline.

## Conclusion

Our results contribute to the growing body of research exploring the complex polygenicity of AD and its association with alterations in functional connectivity at rest and in cognitive functions. Further investigation of the interaction between genetic risk factors and other sociodemographic variables is warranted to understand the epigenetic nature of AD in older adults.

## Data Availability Statement

Data is available upon reasonable request.

## Ethics Statement

The studies involving human participants were reviewed and approved by the Ethics Committee of the Medical Faculty of the Goethe University Frankfurt. The patients/participants provided their written informed consent to participate in this study.

## Author Contributions

AI conducted the data analysis, interpretation of results, and write up of the manuscript. FF was in charge of participant recruitment, data collection, and write up of the manuscript. TMK contributed to the DNA genotyping of participants and calculation of the final individual PRS. TK was involved in the project development, data acquisition and analysis. DP, AR, and JP contributed to designing the study, data collection phase, and revision of the manuscript. EA contributed to the revision of the manuscript. SM was responsible for the conception of the study, study design, supervision of the data analysis and interpretation, and write up and revision of the manuscript. All authors contributed to the article and approved the submitted version.

## Conflict of Interest

The authors declare that the research was conducted in the absence of any commercial or financial relationships that could be construed as a potential conflict of interest.

## Publisher’s Note

All claims expressed in this article are solely those of the authors and do not necessarily represent those of their affiliated organizations, or those of the publisher, the editors and the reviewers. Any product that may be evaluated in this article, or claim that may be made by its manufacturer, is not guaranteed or endorsed by the publisher.
