# Erratum: Investigating the association between polygenic risk scores for Alzheimer's disease with cognitive performance and intrinsic functional connectivity in healthy adults

**DOI:** 10.3389/fnagi.2023.1186490

**Published:** 2023-03-23

**Authors:** Frontiers Production Office

**Affiliations:** Frontiers Media SA, Lausanne, Switzerland

**Keywords:** Alzheimer's disease (AD), genetic disposition, polygenic risk score (PRS), cognition, intrinsic functional connectivity, resting-state fMRI

An omission to the funding section of the original article was made in error. The following sentence has been added: “Open access funding was provided by the Università della Svizzera Italiana.”

The original article has been updated.

