# Supplementary material for: Investigating the Association Between Polygenic Risk Scores for Alzheimer’s Disease With Cognitive Performance and Intrinsic Functional Connectivity in Healthy Adults
Source: Front Aging Neurosci. 2022 May 11;14:837284. doi: 10.3389/fnagi.2022.837284 (PMC9131016; doi:10.3389/fnagi.2022.837284)
Supplement: Supplementary file 1 [file Table_1.pdf]

Table S1: Summary of hierarchical regression analysis of variables predicting performance on working memory tests in the whole group. young group and old group[a]

		Letter Number Span		Spatial Span	
Participants' Group	*Variable*	*ß*	*p*	*ß*	*p*
	Step 1				
Whole Group	*ApoE*	0.05	0.54	0.02	0.83
	Age	-0.36	0.00**	-0.54	0.00**
	Gender	0.15	0.07	0.11	0.13
	Education	0.18	0.04	0.14	0.08
	R^2	0.21		0.34	
	Step 2				
	PRS	-0.05	0.55	-0.15	0.05
	ΔR^2	0.002			
	Step 1				
Young Group	*ApoE*	0.12	0.34	-0.03	0.80
	Age	-0.06	0.64	-0.36	0.00**
	Gender	0.14	0.27	0.02	0.88
	Education	0.15	0.22	0.14	0.22
	R^2	0.05		0.17	
	Step 2				
	PRS	0.08	0.48	-0.09	0.43
	ΔR^2	0.007		0.01	
	Step 1				
Old Group	*ApoE*	0.04	0.58	0.04	0.76
	Age	-0.17	0.19	-0.18	0.15
	Gender	0.17	0.20	0.28	0.04
	Education	0.18	0.17	0.09	0.47
	R^2	0.14		0.16	
	Step 2				
	PRS	-0.27	0.03*	-0.27	0.04*
	ΔR^2	0.08		0.07	

[a] All standardized regression coefficients are from the Step 2 in the analysis. PRS: Polygenic Risk Score. ß: Standardised regression coefficients.

*p < 0.05. **p < 0.01
